# The Treatment of Internal Hæmorrhage by Drugs

**Published:** 1906-03-31

**Authors:** 


### THE TREATMENT OF INTERNAL HÆMORRHAGE BY DRUGS.

In a paper read before the Cambridge Medical Society, Dr. W. E. Dixon[1] analyses the pharmacological actions of various drugs which enjoy a more or less confident repute in the treatment of hæmorrhage, and on the basis of these actions founds a number of practical therapeutic conclusions. He points out that there is a broad contrast between a condition in which application can be directly made to a bleeding point and one in which the seat of hæmorrhage can only be reached by the introduction of remedies into the general circulation. In the one case the position is a very simple one. All that is necessary is to apply to the ruptured vessels substances which either cause clotting of the blood or vascular constriction. As examples of the first of these two groups the tannins and ferric chloride may be mentioned, whilst among drugs which contract the blood-vessels are adrenalin, cocaine, and digitalis. Hence when the source of the hæmorrhage is directly accessible—as in epistaxis, hæmatemesis, bleeding from the uterus, etc.—the hæmorrhage may as a rule be stopped with ease and certainty by the use of one or other of the drugs just mentioned. But the therapeutic problem is much more difficult when the site of hæmorrhage is not accessible to direct medication, and can only be reached by remedies conveyed to it by the circulating blood. For it is possible that before the arrival of the remedy at the bleeding-point it may undergo some transformation by which its therapeutic value is destroyed; or, though exercising a constricting action on the ruptured vessels, this may be neutralised, or more than neutralised, by the increased blood pressure consequent on the contraction due to the action of the drug on the rest of the vascular system. Thus adrenalin introduced either by the stomach or subcutaneously has no vaso-constrictor effect because it is destroyed or modified before it reaches the blood-stream, though a very minute quantity injected into a vein will cause marked contraction of the blood-vessels. Again, the tannins are only very imperfectly absorbed from the alimentary canal, and the form in which they appear in the blood neither causes vascular constriction nor blood coagulation. Thus it is obvious that substances which are very effective in the treatment of a hæmorrhage from the external surface or from a mucous membrane where topical application is possible, may be valueless, or even harmful, in cases of so-called internal hæmorrhage.

There are, however, certain drugs which, when introduced into the blood, will cause certain constriction of the blood-vessels. Ergot, the digitalis group, salts of lead, and some other substances produce this result, and they do so whether administered by the stomach or subcutaneously; whilst adrenalin, as already mentioned, though a powerful vaso-constrictor when injected directly into the blood-stream, has no such effect when given by the mouth or the subcutaneous tissue. Now in considering the application of these vaso-constrictors as possible agents to check internal hæmorrhage, it is necessary to remember that they do not act on all the vessels in equal degree. Thus some vessels are intensely constricted, whilst others, having either no nerve supply or a poor development of muscular tissue in their walls, are almost or entirely unaffected. Hence it may come about that the organs in which the slightly affected vessels exist may actually be congested and their vessels dilated by the administration of a vaso-constricting agent, as the general rise in blood-pressure due to the action of the agent on other vessels may overcome the slight constricting action on the vessels in question. Manifestly this is a consideration of great importance in determining the value of any vaso-constricting drug as a remedy for internal hæmorrhage. For whilst it may be true that the drug constricts the vessels generally, it may in individual organs result in vascular dilatation, and so tend here to promote rather than to stop bleeding. Experiments prove that it is on the vessels of the splanchnic area and of the intestines that the vaso-constrictors exercise their maximum influence, that next in degree of response are those of the skeletal muscles, kidney, spleen, and uterus, whilst in the liver, lungs, brain, and in the coronary vessels there is but a feeble action. The reason for this is suggested by the fact that the pulmonary and coronary vessels receive no nerve supply to their muscles, whilst those of the cerebral and hepatic circulation have at best a very meagre supply.

Applying these principles to individual drugs it is found that adrenalin when injected into the circulation causes great vascular constriction in the splanchnic area and a consequent rise in blood pressure. But so far from there being a similar state of matters in the lungs and brain, the vessels here are dilated and the organs congested by the overplus of blood expelled from the constricted vessels of the splanchnic area. Another point of therapeutic moment in reference to adrenalin is its stimulating effect on the heart, which may be invaluable in tiding over an emergency.

Ergot is another drug which produces intense constriction of the splanchnic vessels and in the vessels of the limbs, but it leads to congestion of the liver, lungs, and meninges. Hence it ought not to be used in cases of cerebral or pulmonary hæmorrhage, but only when the bleeding is from some part of the splanchnic system. In addition to its effect on the peripheral circulation, ergot acts on the cardiac muscle; it increases the vigour of the heart, and augments its output. In this respect it has a more pronounced action than digitalis and deserves more attention from clinicians, though it will hardly gain this until the necessity for the standardisation of its preparations is recognised; most of those at present on the market are unreliable or useless. The rise of blood pressure produced by ergot is much greater than that caused by digitalis and its allies, and, whilst mainly vaso-motor, is partly cardiac. It is also much more sustained than the effect caused by adrenalin; the vascular constriction due to the latter is indeed quite transient, the drug being oxidised in the course of a few minutes.

Of the digitalis group squill is the most effective vaso-constrictor, then follows digitalis, and lastly strophanthus. Hence if constriction of vessels is desired squill is the member of the group which is indicated. Nevertheless, the general effect of squill on the vascular system is similar to that of ergot; the vaso-constriction is seen principally in the splanchnic area, whilst the vessels in the brain and lungs are in a condition of passive dilatation. Much the same must be said of such subtances as veratrine and lead salts, which are occasionally used for their effects on blood-vessels. These undoubtedly cause vascular constriction in the splanchnic area, and hence may be of service for hæmorrhages taking origin therein, though even here their use is hardly desirable. But in the brain, lungs, and liver they lead to passive dilatation of vessels, and are likely therefore to do harm rather than good in hæmorrhage occurring in these organs. The question which follows the above conclusions obviously is, Upon what drugs can reliance be placed in such conditions as hæmoptysis, cerebral hæmorrhage, and hæmorrhage from the liver? First in order of importance is the necessity to avoid any stimulation of the heart or general vaso-constriction, either of which may readily be caused reflexly or by mental disturbance. Hence morphine, by promoting quietness and sleep and by preventing excitement, and by diminishing all sensory reflexes, will probably do more good than any other drug, though it has no direct action on either the heart or the blood-vessels.

It is also possible in the circumstances now under discussion to gain some help by the use of drugs which increase the clotting power of the blood, and of these calcium chloride is of chief value. Given by the stomach, some time must necessarily elapse before this effect is manifest; and as in cases of hæmorrhage the position is usually an urgent one, it is better to give the salt subcutaneously. The calcium chloride should be neutral, and should be injected deeply into the subcutaneous tissue. One or two grains given in this way will diminish the time of blood coagulation in a healthy man to about a half.

[1] Lancet, March 24, 1906.

